# Dietary Quality and Diet-Related Factors Among Emerging Adults (18–23 y) in the United States Are a Cause for Concern: National Health and Nutrition Examination Survey 2015–2018

**DOI:** 10.1016/j.tjnut.2024.06.015

**Published:** 2024-06-27

**Authors:** Alexandra E Cowan-Pyle, Regan L Bailey, Jingjing Gao, Julie M Hess, Lilian O Ademu, Jane Lankes Smith, Diane C Mitchell, Elizabeth F Racine

**Affiliations:** 1 Institute for Advancing Health Through Agriculture, Texas A&M University System, College Station, TX, United States; 2 Department of Management, Policy, and Community Health, University of Texas Health Science Center at Houston, El Paso, TX, United States; 3 Grand Forks Human Nutrition Research Center, United States Department of Agriculture, Grand Forks, ND, United States; 4 Texas A&M AgriLife Research, Texas A&M University, El Paso, TX, United States

**Keywords:** emerging adults, dietary quality, NHANES, Healthy Eating Index, food security

## Abstract

**Background:**

Poor dietary quality is a risk factor for diet-related chronic disease and suboptimal nutritional patterns often begin early in the life course. Although the dietary intakes of young children, adolescents, and middle-aged and older adults are well established, much less is known about emerging adults, who represent a unique time point in life, as they are undergoing significant changes in food environments, autonomy, finances, and caregiver and parental involvement.

**Objectives:**

This study aimed to examine dietary quality, as assessed via the Healthy Eating Index (HEI), by demographic, socioeconomic, and health-related characteristics among emerging adults (18–23 y) in the United States who participated in the 2015–2018 National Health and Nutrition Examination Survey (NHANES).

**Methods:**

NHANES data were collected via a household interview and 2 24-h dietary recalls (24HR). Usual dietary intakes from the 24HRs were approximated using the multivariate National Cancer Institute Method to compute mean HEI-2015 overall and component scores (range: 0–100; higher scores indicating higher dietary quality).

**Results:**

Overall dietary quality among emerging adults (HEI-2015: 50.3 ± 1.3) was significantly lower than other adults (≥24 y) (HEI-2015: 56.3 ± 0.5; *P* < 0.0001) in the United States, with differences primarily driven by lower intakes of whole fruit, vegetables, and whole grains and higher intakes of sodium, refined grains, and saturated fat. Few differences in HEI-2015 scores were noted across population subgroups by sex, food security, family income, and food assistance program participation, except for added sugar; intakes of added sugar were significantly higher among women, food insecure, and food assistance program participants than those in their counterparts, respectively.

**Conclusions:**

Dietary quality is poor among emerging adults in the United States and persists across all population subgroups, suggesting a significant need for tailored public health interventions to improve dietary quality among this population. Future research investigating to what extent emerging adults prioritize healthful behaviors and exploring other indicators for identifying nutritionally vulnerable subgroups may be impactful for identifying disparities among this life stage.

## Introduction

Dietary quality in the United States is derived by adherence to the Dietary Guidelines for Americans (DGA); multiple sources have reported low diet quality across all life stages in the U.S. [1]. Evidence from the 2020–2025 DGA report indicates that adolescents (14–18 y) are most at risk of low diet quality, with similar dietary patterns persisting into younger adulthood [[2], [3]]. Dietary intakes among early adolescents and the general adult population in the United States are well characterized [[4], [5], [6], [7], [8]] compared with those who are entering early adulthood [9,10]. Young adults have been characterized as those aged 19–30 y [1]; however, given the heterogeneity in the context of social and contextual determinants of the variability in eating patterns within this age group [4,[11], [12], [13]], a more tailored investigation into the factors associated with those who are undergoing a dietary transition during a shift from caregiver provision to financial independence, such as those aged 18–23 y (i.e., emerging adults), may be warranted. This population represents an age range with differential food environments, shifts in caregiver/parent involvement and support, financial resource changes, intrapersonal influences, as well as food choice autonomy and procurement of food sources [10,14]. The role of educational and employment status, food access and agency, perceptions of diet and health, and other potentially relevant factors (e.g., disordered eating habits and physical activity) [15,16] remain largely unknown among this age group. For these reasons, associations commonly found in the adolescent or general adult populations may not directly translate to emerging adults specifically.

Given the cumulative effects of health on chronic disease prevention across the life course [1,17], and the concept that this life stage (i.e., emerging adults 18–23 y) may contribute to eating behavior patterns that are carried into later adulthood [10], understanding the various dimensions of health and dietary factors that may influence dietary quality among emerging adults is vital. To that end, identifying critical windows of opportunity where policies and programs can aid in health promotion, optimize nutritional status, and increase equitable access to food and health care resources [18] among emerging adults in the United States is of paramount importance, with potential for lasting effects on nutritional status across the life course.

Thus, this analysis sought to examine dietary quality, as assessed via the Healthy Eating Index (HEI), by several demographic, socioeconomic, and health-related factors, including sex, age, family income, food security status, and food assistance program participation among emerging adults (18–23 y) in the United States who participated in the nationally representative 2015–2018 NHANES. This analysis is intended to generate additional clarity on which factors and in which magnitude may be related to dietary quality in this population subgroup.

## Methods

### NHANES study design

The NHANES is conducted by the CDC National Center for Health Statistics (NCHS) and is a nationally representative, continuous cross-sectional survey of the noninstitutionalized, civilian resident population in the United States [19]. NHANES uses a complex, stratified, multistage probability cluster sampling design and releases publicly available survey data as 2-y datasets [20]. Full details on NHANES data collection and methodologic procedures are described elsewhere [19,20].

For this analysis, demographic, dietary, and socioeconomic and diet-related characteristic data were collected from emerging adults (18–23 y) in the United States who participated in the 2015–2016 and 2017–2018 cycles of NHANES. Participants who were between 18 and 23 y of age, nonpregnant, nonlactating, and determined to be reliable via interviewer assessment [i.e., ≥1 complete and reliable 24-h dietary recall (24HR) per NCHS guidelines] were included in the primary analysis (*n* = 1040). Samples of children (2–17 y; *n* = 4795) and adults (≥24 y; NHANES 2015–2018) in the United States were also used for strictly comparison purposes. All participants or proxies provided written informed consent, and the NHANES protocol (and publicly released deidentified data) was approved by the Research Ethics Review Board at NCHS.

### Demographic, socioeconomic, and health-related characteristics

In NHANES 2015–2018, demographic, socioeconomic, and health-related characteristic data on sex, age, race and Hispanic origin, education, health insurance, self-reported health status, family income, household food security, Supplemental Nutrition Assistance Program (SNAP) participation, alcohol consumption, and smoking status were collected via trained NCHS interviewers using the Computer-Assisted Personal Interview system during the in-home interview. These characteristics (as well as weight status) were evaluated among our primary sample (i.e., those 18–23 y) only. Age in years was classified according to previous emerging adult literature [10,14] and to distinguish the United States emerging adult population (18–23 y) from their child/adolescent (2–17 y) and general adult (≥24 y) counterparts, respectively. Self-reported race and Hispanic origin was grouped by NCHS as non-Hispanic White, non-Hispanic Black, non-Hispanic Asian, and Hispanic or Mexican American. The NHANES “other race” category is included in the overall population-level estimates but is not presented separately. Educational attainment (i.e., highest grade level of education completed) was categorized as follows: less than high school (i.e., less than ninth grade), any high school (ninth to 11th grades, and 12th grade but no diploma), high school graduate or high school equivalency diploma (i.e., GED), or any postsecondary. Classification of health insurance coverage included private and/or public (i.e., inclusive of those enrolled in both private and public health care plans) or uninsured and current self-reported health status categories were as follows: excellent or very good, good, or fair or poor.

Finally, the poverty-to-family income ratio (PIR), defined as the ratio of the annual family income to the poverty guideline set forth by the United States Department of Health and Human Services [21], was used as a measure of family income level and serves as an income eligibility standard for federal nutrition assistance programs. Most notably, a PIR of 130% is an established criterion for potential qualification for SNAP that provides monetary benefits to low-income households for the purchase of foods, with the intention of reducing food insecurity [22]. A PIR of 350% has also been used as a cutoff to distinguish middle-income and high-income households in previous literature [23,24]. For this reason, 3 PIR categories were operationalized in this analysis: PIR ≤ 130% (i.e., SNAP benefit eligibility cutoff); PIR = 131%–350%; and PIR > 350%. Information on food security of the household in the previous 12 months was collected as a component of the interviewer-administered, in-home interview using the USDA’s Household Food Security Survey Module [25,26]. Households classified as having full or marginal food security were considered food secure (i.e., total <3 affirmative responses to questions on food security), whereas those classified as having low or very low food security (i.e., total ≥3 affirmative responses) were considered food insecure [25,26]. SNAP participation status was determined based on whether participants responded “yes” to the question “Do you/Does any member of your household currently receive SNAP or Food Stamp benefits?” Participants were further classified as follows: SNAP participants (i.e., those currently participating), SNAP-income eligible (PIR ≤ 130%) nonparticipants, and SNAP-income ineligible (PIR > 130%) nonparticipants [27,28].

The Alcohol Use Questionnaire (≥18 y) was used to quantify average daily alcohol use in the previous 12 mo, defined according to the frequency of consumption (i.e., number of days in the past 12 mo alcohol was consumed) and the average number of “standard” alcoholic drinks (i.e., 1 drink defined as a 12 oz beer, 5 oz glass of wine, or 1½ oz. of liquor; ∼15 g ethanol) consumed per day; alcohol use was categorized as 0 (i.e., no drinks), 1, 2, or ≥3 drinks/d, in accordance with previous literature [[29], [30], [31]]. Current smoking status was determined via the Smoking—Cigarette Use Questionnaire, according to whether participants classified themselves as never smokers (<100 cigarettes per lifetime), former smokers (>100 cigarettes per lifetime but not current); current—daily smokers; or current—occasional smokers [32].

Weight status, as defined by BMI (in kg/m^2^), was calculated from height and weight measurements during the physical examination in the Mobile Examination Center. Standard BMI categories were as follows: underweight (<18.5), normal weight (18.5–24.9), overweight (25.0–29.9), and obese (≥30.0) [33].

### Dietary assessment

A Dietary Supplement and Prescription Medicine Questionnaire, in tandem with an in-home dietary supplement (DS) product inventory, was administered as a component of the household interview to ascertain information on a participant’s DS use (i.e., all vitamins, minerals, and other DS products consumed) in the past 30 days [34,35].

Following the household interview, self-reported dietary intake data from What We Eat in America, NHANES 2015–2018 were collected via an in-person, 24HR in the Mobile Examination Center as well as a second follow-up 24HR via telephone (∼3–10 d later), using the USDA Automated Multiple-Pass Method [36,37]. Participants reported all foods and beverages consumed from midnight to midnight the previous day for each 24HR. The USDA Food and Nutrient Database for Dietary Studies (FNDDS) [38,39] and the USDA Food Patterns Equivalents Database (FPED) [40] were used to convert all foods and beverages consumed to daily nutrient, food component, and energy intakes. Further details surrounding the Dietary Supplement and Prescription Medicine Questionnaire and 24HR component protocols are available on the NHANES website [34,35,41,42].

### HEI-2015

The HEI-2015 is a scoring metric that can be used to characterize the overall quality of the diet and is designed to measure how closely the dietary intakes of the United States population conform to the 2015–2020 DGA [43,44]. Briefly, the HEI-2015 uses a density-based approach and comprises 13 dietary components, including 9 adequacy (i.e., total fruits, whole fruits, total vegetables, greens and beans, whole grains, dairy, total protein foods, seafood and plant protein, and fatty acids) and 4 moderation components (i.e., refined grains, sodium, added sugars, and saturated fats), with a maximum summary score of 100. In general, higher total HEI-2015 scores indicate higher dietary quality (i.e., range: 0–100); for the adequacy components (i.e., food components to encourage), higher scores reflect higher intakes, whereas, for the moderation components (i.e., food components to limit), higher scores reflect lower dietary intakes [44,45]. Therefore, to score each component in this analysis, reported intake was first linked to the USDA FNDDS and USDA FPED to translate all reported foods and beverages consumed into standardized amounts of foods and nutrients, respectively. Given that this analysis uses the NHANES 2015–2018 survey cycles, the 2015 iteration of the HEI (i.e., that corresponds with the 2015–2020 DGA) is the most up to date iteration of the scale for application within this sample population. The HEI-2015 scoring methodology is described in further detail elsewhere [[43], [44], [45]].

### Statistical analysis

All statistical analyses were performed using SAS (version 9.4; SAS Institute) and SAS-callable SUDAAN (version 11.4; RTI International) software programs; NHANES day 1 dietary sampling weights were applied to account for the NHANES complex survey design and to adjust for differential nonresponse and noncoverage, as well as account for planned oversampling of some population subgroups and poststratification. Descriptive statistics (e.g., prevalence estimates) were approximated using proc surveyfreq, proc surveymeans, and proc descript survey procedures. Standard errors were calculated using Taylor series linearization unless otherwise specified. For demographic and lifestyle characteristics, differences between groups were determined via Satterthwaite-adjusted χ^2^ tests for categorical variables and 2-group *t* tests for continuous variables.

Means and usual intake distributions of HEI-2015 total and component scores were estimated using the multivariate extension of the National Cancer Institute (NCI) Method, which uses a Markov Chain Monte Carlo approach, as described in detail elsewhere [45,46]. Briefly, the Markov Chain Monte Carlo approach is designed to jointly model usual intake distributions of episodically and nonepisodically consumed dietary components simultaneously and account for skewness in observed intake distributions, covariate and nuisance effects on intake (e.g., within-person variation), and modeling correlations among intakes of dietary components. Covariates consisted of day of the week of the 24HR, recall sequence, Dietary Reference Intake age grouping (where applicable), and race and Hispanic origin. SEs for HEI-2015 scores were approximated using Fay-modified balanced repeated replication technique [47,48]. Differences between HEI-2015 total and component scores across population subgroups were compared using 2-group *t* tests; a Bonferroni-corrected *P* value of <0.0167 was considered statistically significant to adjust for multiple comparisons. The publicly available SAS macros for the multivariate extension of the NCI method can be found on the NIH/NCI website [49].

## Results

Emerging adults were aged on average 20.7 y, with a greater proportion of men (54%) than women, and non-Hispanic Whites (51%) than any other race and Hispanic origin group (Table 1). Most demographic and lifestyle characteristics did not significantly differ by sex, as both men and women were generally more likely to have graduated from high school (42%) or have enrolled in postsecondary education (45%), experience food security (74%), have a low or middle family income (74%), reside in a household that was income-ineligible of participating in SNAP (56%), and have private and/or public health insurance (78%) than not. Most emerging adults (86%) self-reported excellent, very good, or good health status, nearly 41% were of normal weight, ∼70% consumed ≥1 alcoholic drink/d, and approximately one-third (∼32%) reported taking ≥1 DS routinely (Table 1).

**TABLE 1:** Demographic and lifestyle characteristics of United States emerging adults (18–23 y): NHANES 2015–2018.^1^

Characteristics	Ages 18–23 y (*n* = 1040)						
	*n*	Total	*n*	Men	*n*	Women	*P*^2^
Sex (%), mean (SE)	—	—	526	53.8 (2.4)	514	46.2 (2.4)	—
Age (y), mean (SE)	1040	20.7 (0.1)	526	20.7 (0.1)	514	20.6 (0.1)	0.6150
Education (%), mean (SE)							0.2334
Less than high school	13	0.8 (0.3)	6	0.8 (0.4)	7	0.8 (0.4)	
Any high school	192	12.4 (1.1)	110	13.2 (1.7)	82	11.4 (1.2)	
High school graduate/GED	412	41.7 (3.5)	208	44.5 (4.3)	204	38.4 (3.9)	
Any postsecondary	423	45.2 (3.1)	202	41.6 (3.6)	221	49.4 (4.0)	
Race and Hispanic origin (%), mean (SE)							0.8514
Non-Hispanic White	296	51.0 (3.2)	139	50.9 (3.9)	157	51.1 (3.5)	
Non-Hispanic Black	237	14.2 (1.7)	125	14.6 (1.9)	112	13.8 (2.2)	
Hispanic or Mexican American	330	25.5 (3.2)	163	25.3 (4.0)	167	25.8 (3.0)	
Non-Hispanic Asian	112	5.6 (0.9)	67	6.0 (1.2)	45	5.1 (1.0)	
Food Security^3^ (%), mean (SE)							0.4866
Food secure	694	73.9 (2.0)	346	74.9 (2.5)	348	72.8 (2.5)	
Food insecure	294	26.1 (2.0)	149	25.1 (2.5)	145	27.2 (2.5)	
Family income (PIR) (%), mean (SE)							0.2654
PIR ≤ 130%	392	37.4 (3.3)	195	36.9 (4.1)	197	38.0 (3.4)	
PIR 131%–350%	345	36.8 (2.8)	160	34.5 (4.2)	185	39.5 (3.2)	
PIR > 350%	178	25.8 (3.1)	98	28.7 (3.7)	80	22.5 (3.6)	
Health insurance (%), mean (SE)							0.8012
Private and/or public	758	77.6 (1.9)	376	78.1 (2.8)	382	77.1 (2.6)	
None	246	22.4 (1.9)	130	21.9 (2.8)	116	22.9 (2.6)	
SNAP participation (%), mean (SE)							0.4287
SNAP participant	238	20.3 (2.3)	105	18.3 (2.5)	133	22.4 (2.8)	
Income-eligible nonparticipant	236	23.9 (3.8)	126	24.7 (4.9)	110	22.9 (3.4)	
Income-ineligible nonparticipant	465	55.9 (2.9)	232	56.9 (3.7)	233	54.7 (3.2)	
Weight status^4^ (kg/m^2^) (%), mean (SE)							0.0769
Underweight	57	5.3 (1.0)	22	3.8 (1.5)	35	7.0 (1.4)	
Normal	437	41.0 (2.0)	238	41.0 (2.7)	199	41.0 (3.2)	
Overweight	230	22.9 (1.9)	130	26.9 (3.1)	100	18.4 (2.1)	
Obese	299	30.7 (2.6)	132	28.3 (3.6)	167	33.6 (3.2)	
Self-reported health status (%), mean (SE)							0.2587
Excellent or very good	427	45.2 (2.7)	242	47.5 (3.4)	185	42.5 (3.3)	
Good	407	40.4 (2.5)	202	40.4 (3.1)	205	40.5 (3.6)	
Fair or poor	169	14.3 (1.7)	69	12.1 (2.3)	100	17.0 (2.7)	
Marital status (≥20 y) (%), mean (SE)							0.0079
Partnered	131	21.7 (2.3)	54	16.5 (2.9)	77	27.9 (3.1)	
Not partnered	419	78.3 (2.3)	225	83.5 (2.9)	194	72.1 (3.1)	
Alcohol use (drinks/d)^5^ (%), mean (SE)							0.0005
0	365	30.0 (1.8)	187	31.7 (2.4)	178	27.9 (2.3)	
1	114	10.9 (1.5)	46	6.2 (1.3)	68	16.4 (2.9)	
2	175	19.4 (1.9)	73	14.7 (2.6)	102	25.0 (2.1)	
≥3	349	39.7 (2.1)	207	47.3 (2.3)	142	30.7 (2.8)	
Smoking status (%), mean (SE)							0.0465
Never	845	76.4 (2.2)	413	73.1 (2.6)	432	80.2 (3.0)	
Former	64	8.6 (1.4)	40	11.6 (2.0)	24	5.2 (1.2)	
Current, occasional	40	4.5 (0.8)	27	5.2 (1.5)	13	3.7 (1.1)	
Current, daily	91	10.4 (1.4)	46	10.0 (1.4)	45	10.9 (2.4)	
Dietary supplement use^6^ (%), mean (SE)	310	31.8 (2.4)	129	27.2 (3.1)	181	37.2 (3.2)	0.0191

Abbreviations: NHANES, National Health And Nutrition Examination Survey; PIR, poverty-to-income ratio; SNAP, supplemental nutrition assistance program.

1 The analytic sample includes individuals 18–23 y who were not pregnant and/or lactating and with complete and reliable information for day 1 and day 2 24-h dietary recalls. NHANES day 1 dietary weights were used to account for the complex survey design of NHANES. Sample sizes in this table vary by demographic and lifestyle characteristic category either owing to missing or unavailable data. Although prevalence estimates reported in this table are nationally representative (i.e., weighted estimates), the sample sizes do not reflect nationally representative estimates but rather the study population. The “other” race and Hispanic origin category (*n* = 65) is not presented in this study but is represented in the overall prevalence estimates.

2 *P* represent Satterthwaite-adjusted χ^2^ tests for categorical variables and 2-sample *t* tests for continuous variables (by sex).

3 Food security status was classified using the USDA Household Food Security Survey Module and according to the overall food security of the household.

4 Weight status is classified according to BMI category for those with complete and available data (underweight: <18.5; normal weight: 18.5–24.9; overweight: 25.0–29.9; obese: ≥30 kg/m^2^).

5 An alcoholic drink refers to 12 ounces of beer, 5 ounces of wine, or 1½ ounces of liquor.

6 Dietary supplement use was classified based on whether the participant responded “yes” to “[taking] any vitamins, minerals, or other dietary supplements in the past month?” on the Dietary Supplement and Prescription Medicine Questionnaire.

Overall dietary quality, as assessed via the HEI-2015, was significantly lower among emerging adults (18–23 y; HEI-2015: 50.3 ± 1.3) than that among the general adult population (≥24 y; 56.3 ± 0.5; *P* < 0.0001) in the United States, but not among children (2–17 y; 52.3 ± 0.6) (Table 2 [44,[46], [47], [48]]). Differences in scores were largely driven by significant differences in HEI component scores, with emerging adults scoring poorly for intakes of whole fruits (HEI-2015 component score: 2.4/5; *P* < 0.007), whole grains (2.0/5; *P* < 0.0046), and sodium (3.3/10; *P* < 0.007) when compared with any other age group. Specifically, when compared with children, intakes of total fruits (2.2 compared with 3.2/5; *P* < 0.0001) and dairy (5.9 compared with 7.5/10 *P* < 0.0001) were significantly lower among emerging adults yet remained suboptimal for both age groups. Similarly, emerging adults also scored lower on total vegetables (3.1 compared with 3.5/5; *P* = 0.0046), refined grains (5.3 compared with 6.6/10; *P* < 0.0001), and saturated fat (4.6 compared with 5.3/10; *P* = 0.0017) than the general adult population (≥24 y) (Table 2 [44,[46], [47], [48]]).

**TABLE 2:** Mean HEI-2015 total and component scores among the United States population, by age group: NHANES 2015–2018^1^

HEI-2015 components	Maximum points	Ages 2–17 y	Ages 18–23 y	Ages ≥24 y
		Mean (SE) (*n* = 4795)	Mean (SE) (*n* = 1040)	Mean (SE) (*n* = 9020)
Adequacy				
Total fruits	5	3.2 (0.1)^a^	2.2 (0.2)^b^	2.5 (0.1)^b^
Whole fruits	5	3.6 (0.1)^a^	2.4 (0.2)^b^	3.0 (0.1)^c^
Total vegetables	5	2.3 (0.1)^a^	3.1 (0.1)^b^	3.5 (0.1)^c^
Greens and beans	5	1.6 (0.1)^a^	2.5 (0.3)^b^	2.9 (0.1)^b^
Whole grains	10	3.3 (0.1)^a^	2.0 (0.2)^b^	2.9 (0.1)^c^
Dairy	10	7.5 (0.1)^a^	5.9 (0.3)^b^	5.2 (0.1)^b^
Total protein foods	5	4.3 (0.1)^a^	4.8 (0.1)^b^	4.8 (0.02)^b^
Seafood and plant protein	5	2.8 (0.1)^a^	3.4 (0.3)^a,b^	4.1 (0.1)^b^
Fatty acids	10	3.2 (0.1)^a^	4.1 (0.3)^b^	4.8 (0.1)^b^
Moderation				
Refined grains	10	4.8 (0.1)^a^	5.3 (0.3)^a^	6.6 (0.1)^b^
Sodium	10	4.9 (0.1)^a^	3.3 (0.2)^b^	3.9 (0.1)^c^
Added sugars	10	6.4 (0.1)^a^	6.7 (0.2)^a,b^	7.0 (0.1)^b^
Saturated fats	10	4.6 (0.1)^a^	4.6 (0.2)^a^	5.3 (0.1)^b^
Total HEI-2015 score	100	52.3 (0.6)^a^	50.3 (1.3)^a^	56.3 (0.5)^b^
Total energy (kcal/d)	—	1848 (18.2)^a^	2141 (89.7)^b^	2133 (13.3)^b^

Abbreviations: HEI, Healthy Eating Index; SE, standard error.

1 Mean HEI-2015 scores were estimated using a multivariate Markov Chain Monte Carlo approach [45,47]. As a result of rounding, HEI-2015 component scores for each group may not add up to their respective total HEI-2015 scores exactly. SEs were approximated using Fay-modified balanced repeated replication technique [48,49], and NHANES day 1 dietary sampling weights were utilized to account for the complex survey design of NHANES. Different superscript alphabet letters (e.g., a, b, c) indicate statistically significant differences in estimates between age groups. A Bonferroni-corrected *P* value of 0.0167 was considered statistically significant. The analytic sample includes individuals ≥2 y who were not pregnant and/or lactating and with complete and reliable information for day 1 and day 2 24-h dietary recalls. HEI-2015 total and component scores were adjusted for family income; household education; food security status; race and Hispanic origin; and age (for 2–17 y and ≥24 y groups only).

While some variation in intakes exist by life stage (i.e., age group), total HEI-2015 scores did not significantly vary by sex among emerging adults in the United States (Table 3 [44,[46], [47], [48]]). In general, component scores were especially low for whole grains (HEI-2015 component scores: 2.0–2.1/10), whole fruits (2.3–2.5/5), total fruits (2.0–2.3/5), greens and beans (2.4–2.6/5), and fatty acids (4.0–4.2/10); however, scores across all adequacy components failed to meet the maximum level, indicating suboptimal intakes for all HEI-2015 adequacy components (Table 3 [44,[46], [47], [48]]). For HEI-2015 moderation components, intakes were especially high for sodium (3.0–3.6/10) and saturated fat (4.4–4.9/10), consistent with the findings observed by life stage. Nonetheless, despite minimal differences in HEI-2015 total and component scores by sex, as compared with women, men scored significantly higher on the HEI-2015 for added sugars (6.1 compared with 7.3/10; *P* = 0.0048) (Table 3 [44,[46], [47], [48]]).

**TABLE 3:** Mean HEI-2015 total and component scores among United States emerging adults, by sex: NHANES 2015–2018^1^

HEI-2015 components	Maximum points	Ages 18–23 y	
		Men	Women
		Mean (SE) (*n* = 526)	Mean (SE) (*n* = 514)
Adequacy			
Total fruits	5	2.0 (0.2)	2.3 (0.2)
Whole fruits	5	2.3 (0.3)	2.5 (0.3)
Total vegetables	5	2.8 (0.2)	3.3 (0.2)
Greens and beans	5	2.6 (0.4)	2.4 (0.4)
Whole grains	10	2.0 (0.3)	2.1 (0.3)
Dairy	10	6.0 (0.4)	5.8 (0.3)
Total protein foods	5	4.9 (0.1)	4.7 (0.1)
Seafood and plant protein	5	3.4 (0.4)	3.5 (0.2)
Fatty acids	10	4.0 (0.4)	4.2 (0.3)
Moderation			
Refined grains	10	5.2 (0.4)	5.5 (0.4)
Sodium	10	3.0 (0.3)	3.6 (0.3)
Added sugars	10	7.3 (0.3)^a^	6.1 (0.3)^b^
Saturated fats	10	4.9 (0.3)	4.4 (0.3)
Total HEI-2015 score	100	50.3 (1.6)	50.3 (1.8)
Total energy (kcal/d)	—	2357 (95.7)^a^	1921 (46.9)^b^

Abbreviations: HEI, Healthy Eating Index; SE, standard error.

1 Mean HEI-2015 scores were estimated using a multivariate Markov Chain Monte Carlo approach [45,47]. As a result of rounding, HEI-2015 component scores for each group may not add up to their respective total HEI-2015 scores exactly. SEs were approximated using Fay-modified balanced repeated replication technique [48,49], and NHANES day 1 dietary sampling weights were used to account for the complex survey design of NHANES. Different superscript alphabet letters (e.g., a, b, c) indicate statistically significant differences in estimates between men and women. *P* values were calculated using 2-group *t* tests; a *P* value of 0.05 was considered statistically significant. The analytic sample includes individuals aged 18–23 y who were not pregnant and/or lactating and with complete and reliable information for day 1 and day 2 24-h dietary recalls. HEI-2015 total and component scores were adjusted for family income; education; adult food security status; and race and Hispanic origin. Age was not adjusted for within this population subgroup given the limited age range.

When evaluated by household food security status, HEI-2015 total and component scores did not significantly differ among emerging adults living with food security or insecurity, with the exception being for added sugars, which were significantly lower (i.e., higher in intake) in terms of HEI-2015 component scores, among emerging adults experiencing food insecurity, than those experiencing food security (Table 4 [44,[46], [47], [48]]).

**TABLE 4:** Mean HEI-2015 total and component scores among United States emerging adults, by household food security status: NHANES 2015–2018^1^

HEI-2015 components	Maximum points	Household food security, mean (SE)	
		Food insecure (*n* = 311)	Food secure (*n* = 677)
Adequacy			
Total fruits	5	2.9 (0.6)	2.2 (0.2)
Whole fruits	5	2.8 (0.5)	2.4 (0.2)
Total vegetables	5	3.3 (0.7)	3.1 (0.2)
Greens and beans	5	2.7 (1.1)	2.7 (0.4)
Whole grains	10	2.0 (0.7)	2.0 (0.2)
Dairy	10	5.9 (0.6)	6.2 (0.3)
Total protein foods	5	4.9 (0.05)	4.8 (0.1)
Seafood and plant protein	5	4.3 (0.5)	3.6 (0.4)
Fatty acids	10	4.3 (0.9)	3.9 (0.3)
Moderation			
Refined grains	10	5.1 (0.6)	5.2 (0.3)
Sodium	10	3.1 (0.7)	3.3 (0.3)
Added sugars	10	5.9 (0.5)^a^	7.0 (0.2)^b^
Saturated fats	10	4.5 (0.8)	4.4 (0.3)
Total HEI-2015 score	100	51.7 (2.8)	51.0 (1.5)
Total energy (kcal/d)	—	2265 (374.8)	2117 (54.8)

Abbreviation: HEI, Healthy Eating Index.

1 Mean HEI-2015 scores were estimated using a multivariate Markov Chain Monte Carlo approach [45,47]. As a result of rounding, HEI-2015 component scores for each group may not add up to their respective total HEI-2015 scores exactly. SEs were approximated using Fay-modified balanced repeated replication technique [48,49], and NHANES day 1 dietary sampling weights were used to account for the complex survey design of NHANES. Different superscript alphabet letters (e.g., a, b, c) indicate statistically significant differences in estimates between adults living in households with food insecurity versus food security. *P* values were calculated using 2-group *t* tests; a *P* value of 0.05 was considered statistically significant. The analytic sample includes individuals aged 18–23 y who were not pregnant and/or lactating and with complete and reliable information for day 1 and day 2 24-h dietary recalls. HEI-2015 total and component scores were stratified by household food security status and adjusted for race and Hispanic origin. Age was not adjusted for within this population subgroup given the limited age range.

As additional parameters of socioeconomic status, HEI-2015 total and component scores were examined by family income and SNAP participation status. Although very few differences were noted across the socioeconomic indicators, low dietary quality persisted; total HEI-2015 scores by family income ranged from 51.0 to 55.3 depending on income level (Supplemental Table 1). Among the HEI-2015 component scores, no significant differences were found, except for total vegetables, with those in the highest income group reporting significantly higher intakes of total vegetables relative to those in the lowest income group (4.5 compared with 2.9/5; *P* = 0.0047) (Supplemental Table 1). Similarly, scores were generally homogenous regardless of whether or not the respondents were participating in SNAP (Supplemental Table 2). Nevertheless, scores varied for seafood and plant protein and added sugars; for seafood and plant protein, income-eligible nonparticipants had significantly higher intakes than income-ineligible nonparticipants, but neither group significantly differed from that of SNAP participants. Additionally, for added sugars, SNAP participants had significantly higher intakes of added sugars when contrasted with their income-eligible and income-ineligible counterparts (Supplemental Table 2).

## Discussion

In NHANES 2015–2018, dietary quality among emerging adults (18–23 y) was significantly lower than that among adults (≥24 y). Poor dietary intakes persisted across all sex, food security, family income, and SNAP participation status subgroups. Although very few differences in HEI-2015 scores were observed by these characteristics, intakes of added sugars were significantly higher among emerging adult women, those living with food insecurity, and SNAP participants, relative to their respective counterparts.

These findings are consistent with a recent analysis by Jun et al. [6] among adolescents (14–18 y) in NHANES 2011–2016 that uncovered that overall diet quality among adolescents was generally poor, with high intakes of added sugars, sodium, and saturated fat, regardless of food security status. The authors indicated that ∼11%–12% of adolescents experienced food insecurity [6], yet this analysis determined that nearly 27% of emerging adults did so. This estimate of food security among emerging adults is not only significantly higher than that of adolescents but also that of children in the United States [50,51] and the general population at the household level [7,52]. According to the USDA Economic Research Service, ∼9% of children experienced household food insecurity in 2022, and nearly 13% of households experienced food insecurity in the same year [53]. Nevertheless, among postsecondary students, the prevalence of food insecurity tends to be much higher [[54], [55], [56]]; the United States Department of Education reported that in 2020, 23% of undergraduates and 12% of graduate students were living with food insecurity [[57], [58], [59]]. This high prevalence of food insecurity among emerging adults is disconcerting not only due to the chronic diet and health implications associated with food insecurity, but also other social determinants that are influenced by nutritional vulnerability, such as educational attainment. Wolfson et al. [56] suggested that experiencing food insecurity during college served as a barrier to time to degree, graduation rates, as well as higher degree attainment, especially among first-generation college students [56].

In a similar manner, while 46% of emerging adults in the United States are income-eligible for SNAP, only ∼25% are enrolled; yet, overall diet did not vary by SNAP participation. In turn, barriers to health-conscious food choices may be present irrespective of SNAP participation status. In addition to increasing awareness, accessibility, and destigmatizing SNAP benefits within this population, encouraging health-conscious food choices among emerging adults through targeted efforts may aid in improving the nutritional status of those in this critical age window; namely, increased opportunities via SNAP-Education (SNAP-Ed) [60], as well as the implementation of tailored public health programs designed to educate those entering early adulthood on nutrition across all sociodemographic subgroups.

Given the instrumental impact of diet and health decisions made in early adulthood on the underpinnings of the diet-health relationship across the life course, understanding these socioeconomic and social determinants, as well as others, that are driving detrimental health behavior patterns among emerging adults is critical for nutritional improvements in this age group. Currently, emerging adults consume a diet that does not align with Federal dietary recommendations and is high in added sugars; when consumed in high quantities, added sugars have been associated with overweight, obesity, and other related comorbidities [1,2]. As such, added sugars were identified as a dietary component of public health relevance in the 2020–2025 DGA for excess consumption [1,2]. Tripicchio et al. [61,62] indicated that for adolescents aged 12–19 y, consuming snacks containing higher amounts of overconsumed dietary components (e.g., added sugar, refined grains, and solid fats) was more common among those with overweight and obesity than their normal weight counterparts; this may be, in part, a pattern that continues into early adulthood given the high prevalence of added sugar intake in this population.

Poor dietary quality at this life stage can establish a negative trajectory for future diet and health quality and has formerly been linked to a number of long-term, adverse health outcomes, such as stroke, cardiovascular mortality, cancer, and neurogenerative diseases [[63], [64], [65], [66]]. The presence of metabolic risk factors during adolescence and early adulthood have been shown to persist into later life [[67], [68], [69]], even with the implementation of lifestyle modifications, and increase risk of premature mortality [70]. Emerging adults are also significantly less likely to take a micronutrient-containing DS than the general adult population in the United States [30,31,[71], [72], [73]] and are a high alcohol user population. These health-related factors, in tandem with high rates of food insecurity, in a period of transition from caregiver provision to financial and personal independence, serve as a cause for public health concern and consideration that differential nutritional programs may be needed to target this high-risk group.

Several caveats should be considered with regard to the present analysis. This study combined 4 y of nationally representative NHANES data to produce statistically reliable estimates that are reflective of the United States population; nevertheless, the small sample sizes for some population subgroup analyses (e.g., by family income, SNAP participation status) may have resulted in larger SEs and a reduced ability to detect statistically significant differences between subgroups. Similarly, owing to the number of statistical tests performed, there is potential for a type I error rate to result from multiple comparisons. Although we did provide exact *P* values in the results section where appropriate to enhance interpretation, study findings should be considered with this limitation in mind. The USDA Automated Multiple-Pass Method is a validated, gold standard method for 24HR data collection, as are the FNDDS and FPED databases used to process the 24HR data [36,37,74]; yet, self-reported dietary data tend to underestimate true energy intake [75]. While random measurement error (i.e., within-person variation in intake) was adjusted for by estimating usual dietary intakes from 2 24HRs using the NCI method (i.e., to compute HEI-2015 scores), systematic measurement error is unable to be fully accounted for and has been previously identified as a potential source of bias with indicators of socioeconomic status [76]. Finally, given the cross-sectional nature of NHANES, temporality and causality are unable to be inferred from this analysis. NHANES provides a wealth of publicly available, nationally representative data designed to monitor the health and nutritional status of the nation; nevertheless, given the vast heterogeneity that exists among emerging adults (e.g., in terms of financial and personal independence), additional data sets that can serve as rich data sources and provide additional context surrounding the independence of those entering early adulthood as well as their differences by geographical location, household composition, and employment status are merited.

In conclusion, emerging adults (18–23 y) in the United States scored a 50.3 of 100 on the HEI-2015, suggesting that dietary quality of this population subgroup is significantly lower than that of the general adult population (≥24 y). These low scores are predominantly driven by low dietary intakes of whole fruit, total vegetables, and whole grains, as well as high dietary intakes of sodium, refined grains, and saturated fat. All these dietary components have been strongly linked with health [1,2]. Similar to adolescents [6], overall dietary quality did not vary by most socioeconomic and demographic characteristics, including sex, food security status, family income, and SNAP participation status.

Encouraging health-conscious behaviors through tailored, precise nutritional interventions in this crucial developmental stage are warranted. Future research should focus on the underconsumed and overconsumed dietary components and food groups identified in this analysis; investigate the heterogeneity across independent, dependent (e.g., on parental/caregiver support), and semi-independent (e.g., college students) emerging adults; examine trends in dietary quality in this population over time; and tailor investigations toward understanding how to promote or limit nutrients and food components of public health relevance within this age group.

## Acknowledgments

We thank Dr. Kelly Fisher and Daniel G. Palmer for critical review and statistical consultation on this manuscript.

## Author contributions

The authors’ responsibilities were as follows – EFR: designed the research and concepts presented; AEC-P, RLB: consulted on the concepts presented and the analytical plan; AEC-P, RLB, EFR: wrote sections of the article; AEC-P: completed the analysis; RLB, JG, JMH, LOA, JLS, DCM, EFR: provided critical review and insights presented; and all authors: read and approved the final manuscript.

## Conflict of interest

Unrelated to this submission, AEC-P is a member of the *Journal of the Academy of Nutrition and Dietetics* Board of Editors. RLB. has served as a consultant in the past to the NIH Office of Dietary Supplements, Nestlé, the General Mills Bell Institute, RTI International, Think Healthy Group, and Nutrition Impact; is a trustee of the International Food Information Council; and is a former board member of the International Life Sciences Institute–North America. RLB has received travel support to present her research on dietary supplements and is a former member of the *Journal of Nutrition* editorial board. EFR has received travel support and stipends for leading Working Groups organized by the Healthy Eating Research Program funded by the Robert Wood Johnson Foundation and the CDC Nutrition and Obesity Policy and Evaluation Network. JG, JMH, LOA, JLS, and DCM report no conflicts of interest.

## Funding

This material is based on the work supported by the USDA, Agricultural Research Service, under Agreement No. 58-3091-1-018. Any opinions, findings, conclusion, or recommendations expressed in this publication are those of the author(s) and do not necessarily reflect the view of the USDA.

## Data availability

Data described in the manuscript is publicly available on the NHANES website. The code book and analytic code will be made available on request pending application and approval.
