# Supplementary material for: Dietary Quality and Diet-Related Factors Among Emerging Adults (18–23 y) in the United States Are a Cause for Concern: National Health and Nutrition Examination Survey 2015–2018
Source: J Nutr. 2024 Jun 27;154(8):2524–33. doi: 10.1016/j.tjnut.2024.06.015 (PMC11826523; doi:10.1016/j.tjnut.2024.06.015)
Supplement: Multimedia component 1 [file mmc1.docx]

**Supplemental Table 1.** Mean Healthy Eating Index – 2015 Total and Component Scores Among U.S. Emerging Adults, by Family Income, NHANES 2015-2018^1,2^

| **HEI-2015 Components** | **Maximum Points** | **Family Income (PIR), Mean (SE)** | | |
| --- | --- | --- | --- | --- |
|  |  | **PIR <130%**  **(n=392)** | **PIR 131-350%**  **(n=345)** | **PIR >350%**  **(n=178)** |
| *Adequacy* |  |  |  |  |
| Total Fruits | 5 | 2.4 (0.6) | 2.4 (0.6) | 2.8 (0.5) |
| Whole Fruits | 5 | 2.4 (0.4) | 2.7 (0.4) | 2.9 (0.6) |
| Total Vegetables | 5 | 2.9 (0.4)^a^ | 3.4 (0.4)^a,b^ | 4.5 (0.4)^b^ |
| Greens and Beans | 5 | 2.2 (1.0) | 3.0 (0.7) | 4.6 (0.4) |
| Whole Grains | 10 | 1.8 (0.6) | 2.3 (0.3) | 1.8 (0.5) |
| Dairy | 10 | 6.3 (0.7) | 6.0 (0.5) | 6.6 (0.8) |
| Total Protein Foods | 5 | 4.9 (0.1) | 4.8 (0.1) | 5.0 (0.03) |
| Seafood and Plant Protein | 5 | 4.1 (0.4) | 3.4 (0.5) | 4.3 (0.5) |
| Fatty Acids | 10 | 4.3 (0.6) | 4.0 (0.6) | 3.8 (0.5) |
| *Moderation* |  |  |  |  |
| Refined Grains | 10 | 5.3 (0.6) | 5.4 (0.4) | 5.0 (0.7) |
| Sodium | 10 | 3.6 (0.7) | 3.7 (0.7) | 2.4 (0.5) |
| Added Sugars | 10 | 6.4 (0.4) | 6.3 (0.5) | 7.6 (0.6) |
| Saturated Fats | 10 | 4.5 (0.7) | 4.5 (0.9) | 4.0 (0.8) |
| Total HEI-2015 Score | 100 | 51.0 (2.3) | 51.7 (2.6) | 55.3 (1.9) |
| Total Energy (kcal/d) | -- | 2221 (193.4) | 2192 (169.9) | 1973 (158.0) |

Abbreviations: HEI, healthy eating index; NHANES, national health and nutrition examination survey; PIR, poverty-to-income ratio; SE, standard error.

^1^ Mean HEI-2015 scores were estimated using a multivariate Markov Chain Monte Carlo approach (44, 46). As a result of rounding, HEI-2015 component scores for each group may not add up to their respective total HEI-2015 scores exactly. Standard errors were approximated using Fay’s modified Balanced Repeated Replication (BRR) technique (47, 48) and NHANES day 1 dietary sampling weights were utilized to account for the complex survey design of NHANES. Different superscript alphabet letters (e.g., a, b, c) indicate statistically significant differences in estimates between PIR groups. *P*-values were calculated using two-group t-tests; a Bonferroni corrected *p*-value of 0.0167 was considered statistically significant.

^2^ The analytic sample includes individuals 18-23y that were not pregnant and/or lactating and with complete and reliable information for the day 1 and day 2 24-hr dietary recalls. HEI-2015 total and component scores were stratified by family income and adjusted for race and Hispanic origin. Age was not adjusted for within this population subgroup given the limited age range.

**Supplemental Table 2.** Mean Healthy Eating Index – 2015 Total and Component Scores Among U.S. Emerging Adults, by SNAP Participant Status, NHANES 2015-2018^1,2^

| **HEI-2015 Components** | **Maximum Points** | **SNAP Participation Status, Mean (SE)** | | |
| --- | --- | --- | --- | --- |
|  |  | **SNAP participant**  **(n=278)** | **Income-eligible nonparticipant**  **(n=217)** | **Income-ineligible nonparticipant**  **(n=441)** |
| *Adequacy* |  |  |  |  |
| Total Fruits | 5 | 2.3 (0.9) | 2.6 (0.5) | 2.6 (0.3) |
| Whole Fruits | 5 | 2.2 (0.7) | 3.0 (0.4) | 2.7 (0.3) |
| Total Vegetables | 5 | 3.0 (0.7) | 3.5 (0.4) | 3.4 (0.3) |
| Greens and Beans | 5 | 2.5 (1.3) | 3.5 (0.6) | 3.0 (0.6) |
| Whole Grains | 10 | 1.9 (0.5) | 2.4 (0.4) | 2.1 (0.2) |
| Dairy | 10 | 6.1 (0.5) | 6.5 (0.7) | 6.0 (0.2) |
| Total Protein Foods | 5 | 4.8 (0.1) | 5.0 (0.03) | 4.8 (0.1) |
| Seafood and Plant Protein | 5 | 3.6 (0.6)^a,b^ | 4.6 (0.3)^a^ | 3.5 (0.3)^b^ |
| Fatty Acids | 10 | 4.2 (1.0) | 4.2 (0.6) | 4.0 (0.3) |
| *Moderation* |  |  |  |  |
| Refined Grains | 10 | 5.1 (0.6) | 5.0 (0.7) | 5.5 (0.5) |
| Sodium | 10 | 3.3 (0.7) | 3.6 (0.5) | 3.1 (0.3) |
| Added Sugars | 10 | 5.2 (0.5)^a^ | 7.1 (0.4)^b^ | 7.2 (0.3)^b^ |
| Saturated Fats | 10 | 4.5 (0.6) | 4.5 (0.9) | 4.3 (0.4) |
| Total HEI-2015 Score | 100 | 48.5 (4.2) | 55.6 (2.1) | 52.1 (1.7) |
| Total Energy (kcal/d) | -- | 2311 (615.2) | 2162 (164.8) | 2107 (62.5) |

Abbreviations: HEI, healthy eating index; NHANES, national health and nutrition examination survey; SE, standard error; SNAP, supplemental nutrition assistance program.

^1^ Mean HEI-2015 scores were estimated using a multivariate Markov Chain Monte Carlo approach (44, 46). As a result of rounding, HEI-2015 component scores for each group may not add up to their respective total HEI-2015 scores exactly. Standard errors were approximated using Fay’s modified Balanced Repeated Replication (BRR) technique (47, 48) and NHANES day 1 dietary sampling weights were utilized to account for the complex survey design of NHANES. Different superscript alphabet letters (e.g., a, b, c) indicate statistically significant differences in estimates between SNAP participation status groups. *P*-values were calculated using two-group t-tests; a Bonferroni corrected *p*-value of 0.0167 was considered statistically significant.

^2^ The analytic sample includes individuals 18-23y that were not pregnant and/or lactating and with complete and reliable information for the day 1 and day 2 24-hr dietary recalls. HEI-2015 total and component scores were stratified by SNAP participation status and adjusted for race and Hispanic origin. Age was not adjusted for within this population subgroup given the limited age range.
